# Integration of Genomic Risk Scores to Improve the Prediction of Childhood Asthma Diagnosis

**DOI:** 10.3390/jpm12010075

**Published:** 2022-01-08

**Authors:** Dilini M. Kothalawala, Latha Kadalayil, John A. Curtin, Clare S. Murray, Angela Simpson, Adnan Custovic, William J. Tapper, S. Hasan Arshad, Faisal I. Rezwan, John W. Holloway, on behalf of STELAR/UNICORN investigators

**Affiliations:** 1 Human Development and Health, Faculty of Medicine, University of Southampton, Southampton SO16 6YD, UK; d.kothalawala@soton.ac.uk (D.M.K.); lpk1r12@southampton.ac.uk (L.K.); W.J.Tapper@soton.ac.uk (W.J.T.); f.rezwan@aber.ac.uk (F.I.R.); 2 NIHR Southampton Biomedical Research Centre, University Hospital Southampton, Southampton SO16 6YD, UK; S.H.Arshad@soton.ac.uk; 3 Clinical and Experimental Sciences, Faculty of Medicine, University of Southampton, Southampton SO16 6YD, UK; 4 Division of Infection, Immunity, and Respiratory Medicine, School of Biological Sciences, Manchester University Hospital NHS Foundation Trust, University of Manchester, Manchester Academic Health Science Centre, Manchester M13 9PL, UK; John.Curtin@manchester.ac.uk (J.A.C.); clare.murray@manchester.ac.uk (C.S.M.); Angela.Simpson@manchester.ac.uk (A.S.); 5 National Heart and Lung Institute, Imperial College of Science, Technology, and Medicine, London SW3 6LY, UK; a.custovic@imperial.ac.uk; 6 The David Hide Asthma and Allergy Research Centre, St. Mary’s Hospital, Isle of Wight PO30 5TG, UK; 7 Department of Computer Science, Aberystwyth University, Aberystwyth SY23 3DB, UK

**Keywords:** asthma, childhood, prediction, polygenic risk score, methylation risk score, machine learning, data integration

## Abstract

Genome-wide and epigenome-wide association studies have identified genetic variants and differentially methylated nucleotides associated with childhood asthma. Incorporation of such genomic data may improve performance of childhood asthma prediction models which use phenotypic and environmental data. Using genome-wide genotype and methylation data at birth from the Isle of Wight Birth Cohort (*n* = 1456), a polygenic risk score (PRS), and newborn (nMRS) and childhood (cMRS) methylation risk scores, were developed to predict childhood asthma diagnosis. Each risk score was integrated with two previously published childhood asthma prediction models (CAPE and CAPP) and were validated in the Manchester Asthma and Allergy Study. Individually, the genomic risk scores demonstrated modest-to-moderate discriminative performance (area under the receiver operating characteristic curve, AUC: PRS = 0.64, nMRS = 0.55, cMRS = 0.54), and their integration only marginally improved the performance of the CAPE (AUC: 0.75 vs. 0.71) and CAPP models (AUC: 0.84 vs. 0.82). The limited predictive performance of each genomic risk score individually and their inability to substantially improve upon the performance of the CAPE and CAPP models suggests that genetic and epigenetic predictors of the broad phenotype of asthma are unlikely to have clinical utility. Hence, further studies predicting specific asthma endotypes are warranted.

## 1. Introduction

It is difficult to reliably identify which symptomatic preschool children will be diagnosed with asthma in later childhood [1,2]. The ability to accurately predict whether a child will be diagnosed with asthma by school age has the potential to support the identification of high-risk individuals who would likely benefit from early intervention, targeted asthma management or preventative strategies. Numerous prediction models for childhood asthma have been reported, with machine learning methods recently emerging as a useful tool to improve upon the performance of models previously developed mainly using traditional statistical methods [3,4]. Whilst some studies applying machine learning methods claim to offer highly accurate models, they often fail to undergo independent validation necessary to promote any future clinical utility of their models [4,5]. We recently applied machine learning approaches to develop two childhood asthma prediction models using clinical and environmental data, the Childhood Asthma Prediction in Early-life (CAPE) and Childhood Asthma Prediction by Preschool-age (CAPP) models [6]. These models outperformed previous validated childhood asthma prediction models, with AUC = 0.71 vs. 0.66 and 0.82 vs. 0.80, respectively, and demonstrated good generalisability when replicated in another UK birth cohort [6].

It is well-established that the consideration of biomarkers for asthma and allergy can facilitate the diagnosis, prediction and identification of asthma phenotypes in addition to showing promise in drug discovery and directing personalised asthma treatment [7,8,9]. Indeed, a number of existing childhood asthma prediction models have incorporated biomarkers in the form of skin prick tests (SPTs) or specific IgE levels to ascertain allergic sensitisation; blood tests to measure eosinophils; or fractional exhaled nitric oxide (FeNO) to provide an indication of airway inflammation in order to improve predictive performance [3].

Over the last few decades, advances in -omic technologies have furthered the investigation of molecular and genomic biomarkers of asthma [10]. Alongside environmental factors, there is a significant genetic component to the susceptibility of developing asthma [11,12,13]. Genetic studies have identified numerous genetic variants associated with asthma [14,15]. Similarly, epigenetic studies have identified CpG dinucleotides whose methylation level is significantly different between asthmatic and non-asthmatic individuals [16,17,18]. The identification of genomic markers able to distinguish between asthmatic and non-asthmatic individuals from large-scale genome-wide and epigenome-wide association studies have the potential to be combined into genomic risk scores that may predict the risk of developing childhood asthma [15,19].

This study aimed to explore whether genomic biomarkers have the potential to improve current predictions of childhood asthma using data from the Isle of Wight Birth Cohort (IOWBC) [20]. For this, the predictive capability of a polygenic risk score (PRS) and two methylation risk scores (MRSs), each accounting for differential methylation patterns observed in newborns (nMRS) and in childhood (cMRS), were assessed independently and upon integration with the CAPE and CAPP models.

## 2. Results

Of the 1456 individuals enrolled in the IOWBC, 1368 individuals had a defined asthma status at age 10 (Table S1; asthma diagnosis was defined as doctor-diagnosis of asthma ever and at least one episode of wheezing or use of asthma medication in the previous 12 months); 924 individuals had genome-wide genotyping data available for 7,236,428 SNPs following standard GWAS quality control; and 765 individuals had genome-wide DNA methylation profiles from Guthrie blood samples (heel pricks performed 7 days after birth) collected for 694,571 probes after preprocessing (see Supplementary Methods).

### 2.1. Polygenic Risk Score

Of the 924 individuals with genotype data available in the IOWBC, 908 individuals had a defined asthma status at age 10 (141 asthmatic and 767 non-asthmatic) and were used to construct the polygenic risk score (PRS).

To construct the PRS (see Supplementary Methods for full details), 128 independent SNPs associated with asthma (annotated to 161 asthma target genes and 47 gene enriched pathways) were considered [21]. Effect sizes from one of the largest GWAS of childhood asthma to date [22] and data in the IOWBC were used to construct the PRS (Table S2).

**Figure 1:**
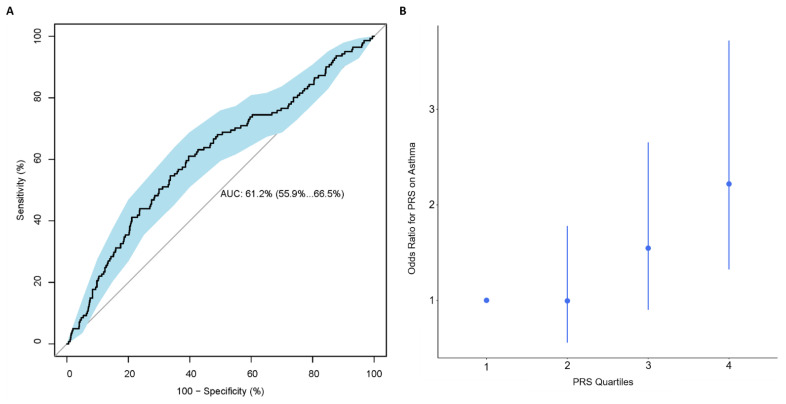
Performance of the best performing childhood asthma PRS in the IOWBC. Subfigures evaluate the best childhood asthma PRS developed in the IOWBC consisted of 105-SNPs (p-value < 0.047). (**A**) ROC curve demonstrating the discriminative ability of the 105-SNP PRS, with 95% confidence intervals calculated from 2000 bootstrapped samples (in blue). (**B**) Quantile plot illustrating the risk of a school-age asthma diagnosis at age 10 across four PRS quantiles (odds ratio +/− 95% confidence intervals with respect to individuals with a PRS in the lowest quartile).

Using PRSice, the best performing PRS consisted of 105 SNPs, calculated using SNPs with *p*-value < 0.047, with R^2^ = 0.027 and AUC (95% CI): 0.61 (0.56, 0.67) (Figure 1A and Figure S1A and Table S3). Based on this 105-SNP PRS, individuals with an asthma diagnosis at age 10 had on average a higher PRS than individuals without asthma (0.13 vs. −0.11, Figure S1B). A quartile plot of all 908 individuals with a PRS calculated in the IOWBC demonstrated a trend effect between an individual’s PRS and their risk of having asthma (Figure 1B); individuals in the highest quartile had over a two-fold increased risk of being diagnosed with asthma at age 10 compared to those in the lowest quartile, with OR (95% CI): 2.22 (1.32, 3.72).

### 2.2. Methylation Risk Scores

Of the 765 individuals in the IOWBC with methylation data collected from Guthrie cards, 747 individuals also had a defined asthma status at age 10 (124 asthmatic, 623 non-asthmatic) and were used to construct each methylation risk score, comparing five different methods currently suggested in the literature (see Supplementary Methods for full details).

#### 2.2.1. Newborn MRS

All six candidate CpGs considered for inclusion in the newborn MRS (nMRS) (see Supplementary Methods) were deemed to be independent—none of the CpGs were highly correlated (Figure S2). Recursive Feature Elimination (RFE) selected all six CpGs for inclusion in the nMRS (Table S4), offering a balanced accuracy score of 0.58. Whilst all five methods used to construct the MRS offered similar discriminative performance, the best nMRS was calculated using MRS 1, with AUC (95% CI): 0.55 (0.50, 0.60) (Table 1).

#### 2.2.2. Childhood MRS

Of the 157 candidate CpGs considered for inclusion in the childhood MRS (cMRS), only one pair of CpGs were found to be highly correlated (*r* = 0.93, Figure S3). Removal of the CpG with the higher *p*-value within this correlated pair of CpGs resulted in 156 independent CpGs for consideration in the feature selection. RFE selected 110 CpGs for inclusion in the cMRS (Table S5), offering a balanced accuracy score of 0.58. All five methods to construct the MRS offered similar discriminative performance (Table 1), however the best cMRS was calculated using MRS 2, with AUC (95% CI): 0.54 (0.49, 0.59).

### 2.3. Integration of Genomic Biomarkers to the CAPE and CAPP Models

**Figure 2:**
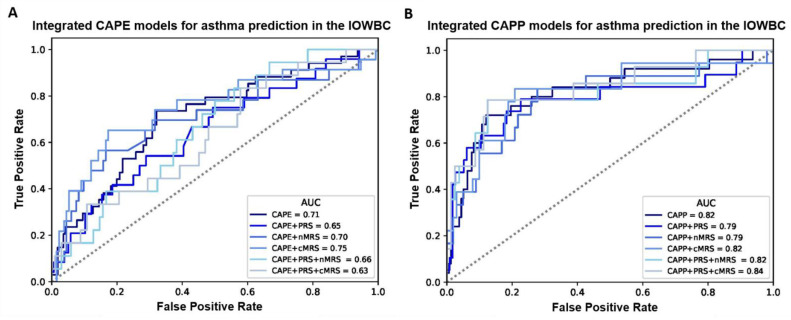
ROC curves comparing the performance of all integrated CAPE and CAPP models in the IOWBC. (**A**,**B**) illustrate changes in the discriminative performance of the CAPE and CAPP models, respectively, upon the integration of the childhood asthma polygenic risk score (PRS) and/or newborn methylation risk score (nMRS) and childhood methylation risk score (cMRS) as additional features in the models.

Overall, for both the CAPE and CAPP models, the integration of the PRS, nMRS or cMRS (either individually or in combination) did not materially improve the discriminative performance of the models in the IOWBC (Tables S6 and S7, Figure 2). However, marginal improvements in model discrimination were observed for the CAPE model upon the integration of the cMRS (AUC: 0.75 vs. 0.71) and the CAPP model upon the integration of both the PRS and cMRS (AUC: 0.84 vs. 0.82).

### 2.4. External Validation

In MAAS, 1018 and 989 individuals had a defined asthma status at ages 8 and 11, respectively (Table S1). Genotype data (for 102 of the 105 PRS SNPs included in the PRS, Table S2) was available among 807/1018 (aged 8) and 767/989 (aged 11) individuals in MAAS. The PRS demonstrated good generalisability to predict an asthma diagnosis, offering a similar AUC as reported in the IOWBC, AUC (95% CI): 0.62 (0.57, 0.68) at age 8 and 0.63 (0.57, 0.69) at age 11.

**Figure 3:**
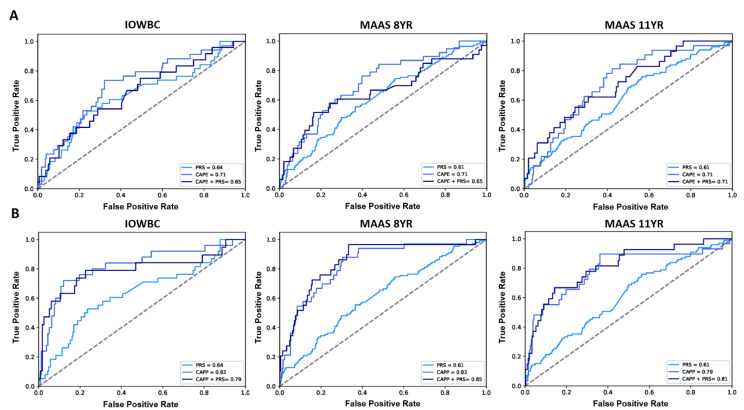
ROC curves comparing the performance of the CAPE and CAPP models integrated with the PRS in the IOWBC and MAAS. (**A**) the discriminative performance of the childhood asthma polygenic risk score (PRS), CAPE model and integrated CAPE model (PRS added as an additional predictor in the model) to predict childhood asthma in the IOWBC at age 10 (left) and the Manchester Asthma and Allergy Study at ages 8 (middle) and 11 (right). (**B**) corresponding information for the CAPP model.

The slight decrease in predictive performance observed upon the addition of the PRS to the CAPE model in the IOWBC (AUC: CAPE = 0.71 vs. CAPE + PRS = 0.65) was replicated in MAAS at age 8 (AUC: CAPE = 0.71 vs. CAPE + PRS = 0.65). When predicting asthma diagnosis at age 11, both the CAPE and integrated CAPE models demonstrated equivalent performance (AUC = 0.71) (Figure 3A). Whilst the addition of the PRS to the CAPP model also resulted in a slight reduction in AUC in the IOWBC (AUC: CAPP = 0.82 vs. CAPP + PRS = 0.79), performance improved when predicting asthma at age 8 (AUC: CAPP = 0.83 vs. CAPP + PRS = 0.85) and 11 years in MAAS (AUC: CAPP = 0.79 vs. CAPP + PRS = 0.81) (Figure 3B).

Due to the unavailability of methylation data from Guthrie blood samples, neither of the MRSs, nor the CAPE and CAPP models integrated with the nMRS or cMRS, could be replicated in MAAS.

## 3. Discussion

Currently, the best validated models for predicting childhood asthma are the CAPE and CAPP models, however they do not consider genomic markers [6]. To account for potential genetic and epigenetic contributors that may further improve the prediction of these models, a PRS and two variations of MRSs for childhood asthma were developed. Individually, the PRS and MRSs demonstrated limited performance. The integration of the genetic and epigenetic risk scores with the CAPE and CAPP models only offered marginal changes in predictive power.

### 3.1. Comparison with Existing Studies

A number of studies have developed genomic risk scores to predict the risk of asthma and other allergic diseases [23,24,25,26]. Specifically for childhood asthma, Spycher et al. constructed a PRS using SNPs known to be associated with childhood asthma (<16 years of age) but was only able to demonstrate modest performance to predict various asthma and wheeze phenotypes (AUC < 0.60) [27]. Belsky et al. showed that individuals at high genetic risk based on a 15-SNP PRS (above the median PRS value) were more likely to develop early-onset childhood asthma (before age 13, hazard ratio [95% CI]: 1.12 [1.01, 1.26]) and experience life-course persistent asthma (onset before age 13 with recurrence up to 38 years, risk ratio (95% CI): 1.36 (1.14, 1.63)) [19]; but while significantly associated, the PRS only offered moderate discriminative ability to predict future asthma (AUC = 0.61). These findings corroborate the moderate predictive performance of the PRS constructed in this study, likely due to the inclusion of similar SNPs—all three PRSs included SNPs annotated to known asthma genes, specifically, the 17q12-21 loci, *IL33* and *IL1RL1* genes.

With asthma being a multifactorial disease, influenced by both genetics and the environment, epigenetic markers which can be both influenced by the environment and genetic variants are of considerable interest [16,17,18]. However, MRSs have not previously been explored for asthma prediction [28]. MRSs developed in other disease areas, including those predicting biological age [29,30] and smoking status [31,32], have demonstrated good predictive potential; however, the performance of the nMRS and cMRS developed in this study was poor (AUC = 0.55 and 0.54, respectively). While the limited performance of the MRSs likely stems from study-specific methodological limitations, it is possible that DNA methylation alone cannot capture the heterogeneity of asthma compared to other disease outcomes. As the MRSs described in this study are the first known MRSs developed for asthma, a robust investigation of the latter explanation was not possible.

Several studies have explored the integration of different data types to improve the prediction of disease outcomes [33,34,35]. For example, predicting lung cancer based on the number of packs smoked per year was significantly improved upon following the addition of either a PRS or MRS, with further improvements following the integration of both genomic biomarkers (improvement in AUC from 0.78 to 0.81, and net reclassification improvement of 14%) [35]. However, only one known study has applied similar integrated predictive modelling for childhood asthma [36]. Dijk et al. used data from the Prevention and Incidence of Asthma and Mite Allergy (PIAMA) cohort to construct a personal and environmental risk score and two PRSs; a 22-SNP PRS based on a multi-ancestry GWAS meta-analysis for asthma conducted by the Trans-National Asthma Genetic Consortium (TAGC), and a 133-SNP PRS based on a GWAS meta-analysis for allergic disease (asthma, eczema or hay fever) conducted within the SHARE consortium. Similar to findings reported in this current study, the integration of neither PRS was able to improve upon the PIAMA personal and environmental score alone (AUC: PIAMA = 0.65; PIAMA + TAGC − PRS = 0.66; PIAMA + SHARE − PRS = 0.65). The lack of predictive improvement offered by childhood asthma PRSs may be explained by the genetic contribution to asthma already being accounted for by predictors included in the clinical models. Whilst this may be a logical explanation for the risk score developed by Dijk et al. which incorporated a predictor of family history, such a predictor was not included in the CAPE or CAPP models. Hence, further investigation is needed.

### 3.2. Selection of Genomic Markers

#### 3.2.1. SNPs Included in PRS

Previous asthma PRSs were developed using single large-scale GWAS studies [19,27,36]. Whilst a gold standard approach would be to use a list of SNPs that have been replicated across a number of GWAS studies, this is not always feasible due to differences in GWAS studies (with respect to sample size, study power, asthma definition and population characteristics) resulting in a limited number of SNPs replicating across studies. Therefore, to avoid limiting findings to a single study, the PRS constructed in this study was based on a curated list of independent SNPs (specific to European populations) summarised across asthma GWASs published until 2019 [21], with effect sizes extracted from a large GWAS specifically for childhood onset asthma [22].

#### 3.2.2. CpGs Included in MRS

Only 12 EWASs for asthma have been conducted to date, of which only two were performed on sample sizes > 150 individuals, suggesting that study power is a key limitation of many existing asthma EWASs [17]. Unlike genotype data which is largely unchanged throughout an individual’s life, methylation levels are changeable, variable across different tissue types and can potentially be reversible in response to different environmental exposures [37]. As no EWAS for childhood asthma to date has used Guthrie blood samples (data available in the IOWBC), the MRSs constructed in this study were based on the prospective newborn EWAS and cross-sectional childhood EWAS meta-analyses performed by Reese et al. using cord blood and childhood blood samples, respectively (the largest asthma EWASs to date) [16]. Therefore, whilst we present the first application of MRSs for childhood asthma, a true determination of the prognostic ability of MRSs will await the completion of larger meta-analyses of EWAS data from relevant samples across multiple cohorts to identify CpG sites consistently associated with childhood asthma.

### 3.3. Methods for Calculating Genomic Risk Scores

The PRS was developed using the clumping-and-thresholding method, a robust method which evaluates the optimal number of SNPs to include in the score based on their GWAS significance (*p*-value) [38]. However, with no gold-standard method yet established for constructing MRSs [28], a number of methods currently proposed in the literature were compared (see Supplementary Material). Calculating the sum of significant CpGs, weighted by their methylation level (beta values) is a method similar to that used to construct the PRS. Indeed, this method gave rise to the best performing nMRS. Interestingly, a simpler scoring method that merely weights CpGs based on whether they are hyper/hypomethylation gave rise to the cMRS with the best discriminative performance. Whilst different methods were selected for the newborn and childhood MRSs, it is important to acknowledge that all MRS methods compared offered similar performance.

### 3.4. Integration of Genomic Markers

To integrate the genomic risk scores with the CAPE and CAPP models, the genetic and methylation risk scores were added as additional predictors to the models and redeveloped using the same training characteristics as the original models. Comparing the performance of the original models and the integrated models with the PRS and/or MRSs, the genomic biomarkers did not appear to offer any substantial predictive benefit, supporting previously reported findings that adding genetic information does not improve the prediction of childhood asthma [36] (previous studies have not integrated methylation data to asthma prediction models). Whilst alternative, potentially more complex, methods of data integration could have been explored, this data integration method was used due to its simplicity and similarity to methods utilised in previous studies. Furthermore, the nMRS and cMRS were not integrated within the same model as this would require blood samples to be collected at two different time-points in a real-world setting, limiting clinical applicability. With the modest performance offered by each MRS individually (and limitations faced in this study), integrating both MRSs with the CAPE and CAPP models (with/without the PRS) was deemed unnecessary and would have been unlikely to have substantially improved model performance.

### 3.5. Strengths and Limitations

This study had a number of strengths. First, the childhood asthma PRS was constructed using a comprehensive list of independent SNPs identified across most asthma GWASs to date, with performance being replicated in an independent population. Second, this is the first known study to construct and evaluate the predictive potential of methylation risk scores for childhood asthma. Third, to our knowledge, this is also the first study to directly compare different methods for constructing MRSs, identifying that all evaluated methods offer equivalent performance to predict childhood asthma. Fourth, the incremental integration of the genetic and methylation risk scores with the CAPE and CAPP models enabled a thorough evaluation of each genomic marker’s predictive capability individually as well as across all different combinations of data aggregation. The generalisability of these integrated models (clinical and genetic data only) was also confirmed in the MAAS cohort, with models demonstrating similar performance to that displayed in the IOWBC.

However, this study did have a number of limitations. First, as GWASs considered by El-Husseini et al. to derive the curated list of asthma SNPs used slightly different asthma phenotype definitions, SNPs less related to the development of childhood onset asthma may have been included in the PRS. Additionally, existing asthma GWASs do not fully account for the heritability of asthma as they are focused on common variants; inclusion of rare variants may capture more of the known heritability of asthma and improve the discriminative ability of future risk scores [39]. Second, methylation data available in the IOWBC (from Guthrie cards collected 7 days after birth) was not directly comparable with methylation data samples used in existing childhood asthma EWASs (cord and childhood blood samples). As a result, the limited performance of the MRSs is likely a reflection of this sample inconsistency rather than a true judgement on the predictive potential of MRSs for childhood asthma. Third, Reese et al.’s EWAS was performed among individuals of mixed ancestry [16]; an EWAS focused on European ancestry may have offered better predictions of childhood asthma in the predominantly Caucasian IOWBC. Fourth, none of the MRSs or MRS-integrated models were able to be replicated as methylation data for relevant CpGs was not available in MAAS. Fifth, data was integrated through the simple addition of the genomic predictors to the feature subsets of the CAPE and CAPP models, and the integrated machine learning models were trained following the same methodology previously used to construct the CAPE and CAPP models [6]. Exploration of alternative data integration approaches is warranted. Such approaches include: (i) a new feature selection including all candidate predictors of the CAPE and CAPP models and the genomic biomarkers; (ii) evaluation of algorithms or class imbalance techniques different to those that gave rise to the CAPE and CAPP models; and (iii) stacked generalization, which aims to construct a meta-model with equal, if not superior, performance to the best performing model consisting of a single data type. Finally, model training using datasets of larger sample sizes, particularly for the models that integrated all data types, may have offered improvements in asthma predictions.

## 4. Materials and Methods

### 4.1. Developmental Study Population

Data was obtained from 1456 individuals enrolled in the Isle of Wight Birth Cohort (IOWBC). Study recruitment and participant details have been previously described [20] (see Supplementary Methods). DNA from peripheral blood samples for 1067 underwent genome-wide genotyping and DNA methylation profiling was performed among 885 individuals from blood samples collected on Guthrie cards 7 days after birth. Genotype data underwent standard quality control and methylation data was preprocessed as previously described [40] (see Supplementary Methods for full details).

### 4.2. Prediction Outcome

School-age asthma diagnosis, evaluated at age 10, was defined as “a doctor diagnosis of asthma ever and at least one episode of wheezing or use of asthma medication in the last 12 months”. Only individuals with a reported asthma status at the 10-year follow-up were included in the analyses (*n* = 1368).

### 4.3. Construction of the PRS

To construct the PRS, 128 independent SNPs found to be associated with asthma in European populations by El-Husseini et al. were considered (see Supplementary Material) [21]. Summary statistics for the 128 SNPs were extracted from a single GWAS study recently conducted by Ferreira et al. [22] The childhood asthma PRS was constructed using the clumping and thresholding method using PRSice [41] and calculated as the sum of an individual’s risk alleles weighted by the allele effect size (estimated from the GWAS) for each SNP. The best PRS from all the scores calculated from the thresholding method was selected as the score which offered the highest AUC across 2000 bootstrapped samples (see Supplementary Material).

### 4.4. Construction of the MRS

Two MRSs—a newborn MRS (nMRS) and childhood MRS (cMRS)—were constructed using significant CpGs identified from Reese et al.’s prospective and cross-sectional EWAS meta-analyses for childhood asthma (7–17 years), respectively (see Supplementary Material) [16].

Correlation between CpGs were assessed using Spearman’s rank correlation and highly correlated CpGs were excluded. Independent CpGs for each MRS then underwent feature selection using recursive feature elimination (RFE), using a random forest algorithm, to identify the optimal subset of CpGs to include in each MRS. Five different methods for calculating MRSs reported in the literature were compared and the best score was selected as the method which offered the highest AUC across 2000 bootstrapped samples (see Supplementary Material).

### 4.5. Integration of the Genomic Biomarkers with the CAPE and CAPP Models

As previously described [6], the Childhood Asthma Prediction in Early-life (CAPE) and Childhood Asthma Prediction at Preschool age (CAPP) support vector machine algorithms use 8 and 12 clinical, demographic and environmental predictors of childhood asthma available from the first two and first four years of life, respectively.

The genomic biomarkers were integrated with the CAPE/CAPP models in a stepwise manner, whereby the following models were developed: (i) CAPE/CAPP plus PRS; (ii) CAPE/CAPP plus nMRS; (iii) CAPE/CAPP plus cMRS; (iv) CAPE/CAPP plus PRS and nMRS; and (v) CAPE/CAPP plus PRS and cMRS. Data was integrated by adding the relevant genomic risk scores as additional predictors to each model’s existing feature set. The integrated models were then trained using the same algorithm and training dataset characteristics which obtained the CAPE and CAPP models (see Supplementary Methods).

### 4.6. Evaluation of Model Performance

Model performance was evaluated based on the discriminative performance in the IOWBC holdout test set using the area under the receiver operating characteristics curve (AUC). Sensitivity, specificity, positive and negative predictive values (PPV and NPV), positive and negative likelihood ratios (LR+ and LR−), balanced accuracy and F_1_-score were reported at the optimal threshold that maximized the Youden’s Index, with 2000 bootstrap samples used to calculate the 95% confidence intervals for each performance measure.

### 4.7. External Validation

The generalisability of the genomic risk scores and the integrated CAPE and CAPP models was assessed in the unselected Manchester Asthma and Allergy Study (MAAS) cohort, which has been previously described [42]. Only individuals with data available for all of the predictors in each model were used in the external validation to predict school-age asthma at ages 8 and 11 (see Supplementary Material).

## 5. Conclusions

Using data integration approaches, novel genomic risk scores (a PRS and two MRSs) for childhood asthma were developed and combined with the current best performing validated childhood asthma prediction models, the CAPE and CAPP models. Each genomic risk score offered limited predictive performance individually and were unable to substantially improve upon the performance of the CAPE and CAPP models (developed using demographic, clinical and environmental data alone) upon integration. These findings suggest that genomic biomarkers are less important in the prediction of childhood asthma once the impact of a child’s environment has been accounted for, particularly epigenetic risk factors which are known to be influenced by changes in environmental factors. However, with the complex interplay between environmental, genetics and epigenetic risk factors in asthma development still being uncovered, these genomic components cannot be dismissed [15]. Moreover, in line with ongoing efforts to untangle the heterogeneity of asthma [9], future research developing genomic risk scores in larger cohorts stratified for specific asthma phenotypes and endotypes, using common methodologies for data collection, are warranted. Consideration of other -omic data, such as transcriptomics and metabolomics, may also improve the predictability of childhood asthma diagnoses [43]. Nevertheless, despite their current limited predictive capabilities, genomic biomarkers for predicting childhood asthma may offer useful research insights. For example, extending research beyond gene-environment interactions, and further exploring PRS-environment interactions, may uncover non-linear effects of environmental risk factors and subsequently identify patient subgroups that may benefit from precision medicine [15,44]. Consideration of such interaction effects as predictors in future models could potentially improve childhood asthma predictions.

## Figures and Tables

**Table 1:** Performance of the newborn and childhood methylation risk scores calculated using five different methods.

MRS Method ^†^	Newborn MRS: 6 CpGs AUC (95% CI)	Childhood MRS: 110 CpGs AUC (95% CI)
1	0.55 (0.50–0.60) ^‡^	0.53 (0.48–0.59)
2	0.54 (0.48, 0.59)	0.54 (0.49, 0.59) ^‡^
3	0.49 (0.44, 0.55)	0.53 (0.48, 0.59)
4	0.53 (0.48, 0.59)	0.53 (0.48, 0.59)
5	0.52 (0.47, 0.58)	0.53 (0.48, 0.59)

^†^ Full descriptions of the MRS methods are detailed in the supplementary material. ^‡^ Scores offering the best discriminative performance, and which were used in subsequent analyses.

## Data Availability

The data that support the findings of this study are available on request from the corresponding author. The data are not publicly available due to privacy or ethical restrictions.

## Supplementary Materials

The following are available online at https://www.mdpi.com/article/10.3390/jpm12010075/s1, Full descriptions of the datasets and methods used; Figure S1: Evaluation of the best performing childhood asthma PRS in the IOWBC, Figure S2: Correlation matrix of candidate CpGs considered for the newborn MRS, Figure S3: Correlation matrix of the 22 nearby CpGs considered for the childhood MRS, Table S1: Distribution of the CAPE and CAPP model predictors for all individuals in the IOWBC and MAAS with a defined asthma status at each timepoint, Table S2: Candidate SNPs considered for inclusion in the PRS, Table S3: Performance of all evaluated PRSs, Table S4: Candidate CpGs considered for inclusion in the newborn MRS, Table S5: Candidate CpGs considered for inclusion in the childhood MRS; Table S6: Performance of all integrated CAPE models in the IOWBC and MAAS, Table S7: Performance of all integrated CAPP models in the IOWBC and MAAS.
