# Supplementary material for: Integration of Genomic Risk Scores to Improve the Prediction of Childhood Asthma Diagnosis
Source: J Pers Med. 2022 Jan 8;12(1):75. doi: 10.3390/jpm12010075 (PMC8777841; doi:10.3390/jpm12010075)
Supplement: Supplementary file 1 [file jpm-12-00075-s001.zip › Supplementary Material.pdf]

Supplementary Material

Datasets

Isle of Wight Birth Cohort (IOWBC)

The IOWBC, also known as the second generation (F_1) cohort, is based on the Isle of Wight (IoW), off the south coast of England, UK. It is a whole population, single-centre prospective cohort study established in 1989 to explore the natural history and development of asthma and other allergic diseases in early life[1]. Between 1989 and 1990, 1509 women gave birth to 1536 children on the Isle of Wight. From these births, 1456 children were recruited into the IOWBC study and followed up from birth and at 1, 2, 4, 10, 18 and 26 years, with high retention rates of 94.0%, 84.5%, 83.7%, 94.3%, 90.2% and 70.9%, respectively[1]. The IOWBC cohort comprises of individuals predominantly of Caucasian ethnicity (98%).

Demographic, clinical and environmental data

In the IOWBC, allergic disease and exposure-related data was collected through hospital records, physical examinations and study specific questionnaires. From the 10 year follow-up, questionnaires were standardised with the International Study of Asthma and Allergies in Childhood (ISAAC) questionnaire created in 1995[2].

At each follow-up, demographic and lifestyle information as well as pregnancy and birth characteristics, environmental exposures and indicators of asthma and allergy status were collected (total number of variables: at birth=70; 1-year=124; 2-year=110; 4-year=115; 10-year=306; 18-year=430; 26-year=460). Specifically, this included data on family history; gestational factors, breastfeeding and early life diet; household pets; exposure to tobacco smoking; housing characteristics; socioeconomic status as well as height, weight and BMI. Clinical symptoms for which data was collected included: wheeze, cough, nasal symptoms, nocturnal symptoms, chest infections, eczema, allergic rhinitis, food allergy and asthma. In addition, a skin prick test (SPT) was performed in infants with allergy-related symptoms at 1 and 2 years, and in all participants from the 4-year follow-up onwards. Sensitisation to the following 13 common inhaled and food allergens was assessed: house dust mite (*Dermatophagoides pteronyssinus*), grass pollen mix, tree pollen mix, cat and dog epithelia, *Alternaria alternata*, *Cladosporium herbarium*, milk, hen's egg, soya, cod, wheat, and peanut. A positive SPT was confirmed if the mean wheal diameter was at least 3mm greater than the negative control (physiologic saline). An individual was considered atopic following at least one positive SPT.

Genotype data

Blood samples were collected from cohort members at multiple time-points - from heel pricks at 7 days of age (collected on Guthrie cards) and at 10, 18 and 26 years. Peripheral blood samples collected at the 18-year time-point were primarily used to extract DNA for genotyping; where unavailable, blood samples collected at age 7 days, 10 years or 26 years were used. DNA from the peripheral blood samples of 1067 individuals in the IOWBC underwent genome-wide genotyping using the Illumina InfiniumOmni2.5-8v1.3 microarray. Standard quality control for genome-wide association studies (GWAS) was performed to exclude samples with low call rate (<97%) and SNPs with call rate <95%, minor allele frequency (MAF) <0.005 and significant deviation from Hardy-Weinberg equilibrium (p-value $<1x10^{-8}$) prior to imputation. Alleles had also been updated to match the direction (forward) and coordinates of the reference dataset, GRCh37[3]. Data were pre-phased

(EAGLE2)[4] and imputed (PBWT)[5] using the Sanger Imputation Service (https://www.sanger.ac.uk/tool/sanger-imputation-service).

Further quality control was performed to prepare the imputed genotype data for the analyses conducted in this study. This included the retention of data with an imputation quality >80%. SNPs were again filtered to exclude those with call rate <95%, MAF<0.01 and significant deviation from Hardy-Weinberg equilibrium (p-value $<1x10^{-6}$). Samples were further filtered to remove those with call-rate <97%, extreme heterozygosity (±3SD of the mean F-coefficient) and gender mismatch. One individual of each related pair (3rd degree relations or closer, pi-hat ≥0.125) was also excluded. Population structure was assessed by principal component analysis (PCA), comparing the IOWBC with the European descent (CEU), Yoruba (YRI), Hans Chinese (CHB) and Japanese (JPT) HapMap3 reference populations[6]. Non-European individuals were excluded based on visual inspection of a PCA plot. A final dataset of 977 individuals with genotype data for 7,236,427 SNPs was retained for downstream analyses.

In addition, five variants of the filaggrin gene (R501X, 2282del, S3247X, 3702delG and R2447X) had previously undergone genotyping in the IOWBC using GoldenGate Genotyping Assays (Illumina, Inc, SanDiegom CA) on the BeadXpress Veracode platform per Illumina's protocol[7]. In brief, 1,248 blood samples (from 1,211 individuals and 37 replicates) were fragmented, hybridised to allele-specific primer sets and subject to extension/ligation reactions. Samples were sourced from a similar mixture of time points as described for the genome-wide genotype data. Samples were then hybridised to the Veracode bead pool for processing by the BeadXpress reader. Allele determination was based on a GenCall score >0.25. Scores below this quality threshold were deemed "no calls".

For downstream analyses conducted in this study, quality-controlled genotype data for the R501X variant of the filaggrin gene (n=924) was added to the quality controlled imputed genome-wide genotype profiles detailed above, resulting in a final dataset of 924 genotype profiles consisting of 7,236,428 SNPs.

Methylation data

Genome-wide DNA methylation data was measured at birth from blood samples collected on Guthrie cards (n=885). DNA from Guthrie cards was extracted using the Gensolve kit, following the procedure described by Beyan *et al.*[8]. 500ng of isolated genomic DNA from each sample was then bisulphite-treated using the EZ 96-DNA methylation kit (Zymo Research, Irvine, CA, USA). DNA methylation profiling was then performed using the Illumina Infinium MethylationEPIC BeadChips following the manufacturer's standard protocol. In this process, the DNA methylation levels at 863,904 CpG sites were estimated as beta values - the ratio of the methylated probe intensity and the overall intensity. DNA methylation beta values range from 0 (completely unmethylated CpG) to 1 (completely methylated CpG). Due to funding limitations, Guthrie DNA methylation data was collected and profiled (sent to the same service provider) in seven batches.

DNA methylation data underwent a number of pre-processing steps as previously described[9]. Briefly, beta values were normalised using CPACOR[10]. Illumina Background Correction was applied to intensity values prior to the exclusion of CpGs with intensity values with detection p-values $\geq 10^{-16}$ and samples with call-rate <95%. Using the minfi package[11], gender was inferred based on the difference in median total intensity of CpGs on the X and Y chromosomes. Individuals whose predicted gender directly contradicted their reported gender, as well as those who deviated ±4SD from the main gender clusters, were excluded. One individual of each set of repeat sample and related pair of individuals (3rd degree relations or closer, pi-hat ≥0.125) were retained. Quantile normalisation was applied to intensity values using the DASEN method[12], incorporating control

probe adjustment and global correction reduction. As DNA methylation was measured in seven batches, batch effects were removed using ComBat (sva package)[13]. SNP-associated and cross-hybridised probes were removed[14]. A total of 765 individuals with DNA methylation profiles consisting of 694,571 CpGs were retained for further analyses.

MAAS

Clinical data

The Manchester Asthma and Allergy Study (MAAS) is an unselected birth cohort established to study the development of asthma and other atopic disorders in childhood[15]. Parents were recruited into the study from 50 square miles of South Manchester and Cheshire (within the maternity catchment area of the Wythenshawe and Stepping Hill Hospitals). Between 1995 and 1997, 1211 women (≤10 weeks pregnant) were recruited into the study and 1184 children were subsequently followed up at 1, 3, 5, 8, 11, 13-16 and 18 years. The MAAS cohort consists of a stable mixed urban-rural population (~89% Caucasian).

Medical records and validated questionnaires were used to collect data on clinical symptoms of allergy and asthma and environmental exposures[15,16]. Blood samples were taken and SPT and lung function tests were performed from the three-year follow-up onwards. SPT for house dust mite (*Dermatophagoides pteronyssinus*), cat, dog, grass pollen, moulds, milk, and egg were performed from the 3-year follow-up onwards; tree pollen and peanut allergens were also tested from the 8-year follow-up onwards. A mean wheal diameter at least 3mm greater than the negative control (physiologic saline) was used to confirm a positive SPT. An individual was considered atopic following at least one positive SPT.

Early life predictor data was collected from the 1-year and 3-year follow-ups in MAAS (1-year and 2-year follow-ups in the IOWBC), and from the 5-year follow-up for preschool predictors (4-year follow-up in the IOWBC).

Genotype data

DNA samples were genotyped using the Illumina 610 quad chip. Genotypes were called using the Illumina GenCall application following the manufacturer's instructions. Prior to imputation samples were excluded on the basis of gender mismatches; minimal or excessive heterozygosity, genotyping call rates of <97%. SNPS were excluded if they had call rates of <95%, minor allele frequencies of <0.5% and HWE $p<3x10^{-8}$. Prior to imputation each chromosome was prephased using EAGLE2 (v2.0.5) as recommended by the Sanger Imputation Server. We then imputed data from 919 samples with PBWT with the Haplotype Reference Consortium (release 1.1) of 32,470 reference genomes using the Sanger Imputation Server.

Imputed genotype data was further quality controlled to exclude SNPs with INFO<0.80, MAF<0.01 and deviations from HWE (p-value $\leq 1\times10^{-8}$). One individual from each pair of siblings or cryptic relations and non-Caucasians were also excluded. A final dataset 102 SNPs from 852 individuals were used to generate polygenic risk score in MAAS.

Methods

Construction of the PRS

Candidate predictors for the PRS

To construct the PRS, 128 independent SNPs associated with asthma (annotated to 161 asthma target genes and 47 gene enriched pathways) were considered. These SNPs were identified from a recent study conducted by El-Husseini *et al*. which provided an updated summary of independent SNPs associated with asthma from published GWASs between 2007 and 2019[17]. In brief, SNPs with genome-wide significance ($p<3x10^{-8}$) were identified and tested for independence in European populations using the LDmatrix tool on LDlink[18]. The study considered SNPs to be independent if the linkage disequilibrium (LD) correlation (r^2) was less than 0.05.

The list of 128 SNPs was summarised from a combination of different asthma GWASs. Therefore, to construct the PRS specifically for childhood asthma, summary statistics for the 128 SNPs were extracted from a single GWAS study recently conducted by Ferreira *et al*.[19]. This was the largest GWAS to identify SNPs associated with the most relevant childhood onset asthma phenotype that used data from UK Biobank (similar population to the IOWBC and MAAS). SNPs were included in the construction of the PRS if genotype data (after standard GWAS quality control) was available in the IOWBC and summary statistics were available in Ferreira *et al*.'s GWAS. Where data for a SNP was unavailable in either the IOWBC or GWAS summary statistics, the closest proxy SNP in high LD ($r^2>0.80$) within the European British in England and Scotland (GBR) population, with data available in the IOWBC, was sourced using the LDproxy tool on LDlink[20].

The summary statistic data extracted for each SNP from the GWAS included: i) effect size - to weight each SNP in the PRS, and ii) p-value - to determine the inclusion of SNPs in the score using the thresholding method. Where proxy SNPs were used, the effect size and p-value of the original SNP were used.

Calculation of the PRS

The childhood asthma PRS was constructed using the clumping and thresholding method using PRSice[21]. For this method, clumping is first performed to ensure that the SNPs which will be included in the PRS are independent by grouping nearby SNPs together and removing those in high LD. Next, thresholding is the process in which SNPs are included into the score based on whether they are below a pre-specified p-value threshold. Using the thresholding method, a number of scores are constructed across a range of p-value thresholds and the best score is selected based on specified criteria. By default, PRSice performs clumping and removes SNPs in high LD ($r^2>0.1$) within a 250kb window and calculates scores across all possible p-value thresholds.

The PRS was calculated as the sum of an individual's risk alleles weighted by the allele effect size for each SNP as estimated from the GWAS study (Equation 2).

$$PRS = \sum_{i}^{N} X_i \beta_i$$

Equation 2 Formula for calculating a weighted polygenic risk score

The PRS is calculated as the sum of all SNPs included in the score (*N*). For each SNP in the PRS (*i*), the dosage of the risk allele (*X*) is weighted by its GWAS effect size estimate (*β*).

To select the best PRS from all the scores calculated from the thresholding method, PRSice uses Nagelkerke's R^2 goodness of fit statistic which evaluates how well the score explains the variance in a binary phenotype. Whilst Nagelkerke's R^2 was considered, in line with the selection of the best CAPE and CAPP models in Kothalawala *et al*., the final PRS was selected as the score which offered the highest AUC across 2000 bootstrapped samples.

Construction of the MRS

Candidate predictors for the MRS

To construct the MRS in the IOWBC, CpGs significantly associated with childhood asthma were extracted from a recent EWAS meta-analysis for childhood asthma published by Reese *et al*.[22]. In this study, two separate EWAS meta-analyses for childhood asthma (7-17 years of age) were performed. The first was a prospective EWAS which used DNA methylation data from cord blood samples (newborn EWAS) whilst the second was a cross-sectional EWAS which used peripheral blood samples collected between 7-17 years (childhood EWAS). Two MRSs – a newborn MRS (nMRS) and childhood MRS (cMRS) - were constructed using significant CpGs identified from each EWAS meta-analysis. Of the 9 CpGs found to be significantly associated with asthma from the newborn EWAS, data for only 6 CpGs was available in the IOWBC after pre-processing of the methylation data. For the childhood MRS, the EWAS identified 164 CpGs associated with asthma, of which 157 has data available in the IOWBC.

To ensure that only independent CpGs were included in each MRS, the correlation between the CpGs considered for each model were evaluated. Due to the skewed distribution at some CpGs, Spearman's rank correlation coefficient was used to evaluate correlation between CpG sites (CpGs with $R^2>0.8$ were considered highly correlated). Independence between CpGs was also evaluated based on the distance between CpGs and their regional positions if found within the same CpG island; studies have identified that nearby CpGs (<2000 base pairs from each other) are often co-methylated and CpGs found within the same region of a CpG island are suggested to be non-independent[23,24]. Where correlated pairs of CpGs were identified, the CpG with the higher p-value reported from the EWAS meta-analysis was discarded.

A feature selection of the independent CpGs considered for each MRS was then performed by Recursive Feature Elimination (RFE) using a random forest algorithm within a 5-fold cross validation. For feature selection, the beta values for each CpG were first standardised. In line with the feature selection performed by Kothalawala et al. in the construction of the CAPE and CAPP models[25], the optimal subset of CpGs to include in each MRS was selected based on the average balanced accuracy score within a stratified five-fold cross-validation.

Calculation of the MRS

As no gold-standard method has yet been established for calculating MRSs[26], five different calculations identified from the literature were compared (Equation 3-7). For each MRS, the best score was selected as the calculation which offered the highest AUC across 2000 bootstrapped samples.

$$MRS\ 1 = \sum_{i}^{N} \beta_i w_i$$

Equation 3 MRS 1

Algorithm as reported by Fernandez-Sanles *et al.*[27]. Score is calculated as the sum of the beta value (β) multiplied by the effect size as reported in the EWAS meta-analysis (w), for all CpGs included in the score (N).

$$MRS\ 2 = X\ \{X\ \in M\}$$

Equation 4 MRS 2

Algorithm as reported by Guan *et al.*[28]. Score is calculated as the count (#) of the number of CpGs (X) that were hypermethylated or hypomethylated (M). A CpG was considered hyper (hypo)-methylated if methylation levels were in the upper (lower) quartile of the distribution among controls.

$$MRS\ 3 = \frac{1}{N}\sum_{i}^{N} w_i (\frac{\beta_i - \mu_c}{\sigma_c})$$

Equation 5 MRS 3

Algorithm as reported by Yu *et al.*[29], where N denotes the number of CpGs considered in the MRS, β is the methylation (beta) value of the CpG and μ_c and σ_c are the mean methylation (beta) value and standard deviation among non-asthmatic controls, respectively. Each CpG in the score was weighted (w), with hyper(hypo)methylated CpGs assigned a weight of +1(-1).

$$MRS\ 4 = \frac{1}{N}\sum_{i}^{N} w_i \left(\frac{\beta_i - \mu_c}{\sigma_c}\right)$$

Equation 6 MRS 4

Algorithm as reported by Yu *et al.*[29], where N denotes the number of CpGs considered in the MRS, β is the methylation (beta) value of the CpG and μ_c and σ_c are the mean methylation (beta) value and standard deviation among non-asthmatic controls, respectively. Each CpG in the score was weighted (w) using the effect size reported in the EWAS meta-analysis.

$$MRS\ 5 = \sum_{i}^{N} \frac{\beta_i}{\beta_N}(Y)$$

Equation 7 MRS 5

Algorithm as reported by Elliot *et al.*[30], where β_i is the methylation (beta) value of each CpG, β_N is the average effect size across all CpGs and Y denotes the difference between the CpG methylation value and the median methylation level reported among non-asthmatic controls (reference methylation beta value). For CpGs associated with an increased methylation level in asthmatics, Y= beta value –

reference methylation beta value. For CpGs associated with a decreased methylation level in asthmatics, *Y*= reference methylation beta value –beta value.

Integration of the genomic biomarkers with the CAPE and CAPP models

CAPE Model

As previously described by Kothlawala et al. [25], the CAPE model comprised of 8 predictors selected through a recursive feature elimination process from a list of 39 candidate predictors available in the IOWBC between birth and early life (a combination of the 1 and 2 year follow-ups). These predictors were: maternal age at the time of the child's birth, maternal socioeconomic status (SES), birthweight, total breastfeeding duration, age of solid food introduction, BMI at 1 year, early life wheeze, and early life cough.

765 individuals had complete data for these predictors and had a defined asthma outcome at age 10 in the IOWBC. Following a 2:1 stratified split of the data into a training and hold-out validation set, a support vector machine (SVM) algorithm was trained on the training dataset, undersampled to balance class proportions. Hyperparameters for the SVM (with parameters kernel=radial basis function, C=4.5.1, gamma=0.0054) were identified through a grid search within a 5-fold cross validation framework.

CAPP Model

Based on a list of 54 candidate predictors available in the IOWBC from birth, early life (1 and 2 year follow ups combined) and preschool age (4-year follow up), RFE selected 12 predictors for inclusion in the CAPP model. These predictors were: maternal age at the time of the child's birth, maternal SES, birthweight, total breastfeeding duration, age of solid food introduction, BMI at 1 year, BMI at 4 years, preschool wheeze, preschool cough, preschool nocturnal symptoms, preschool atopy and preschool polysensitisation status[25].

548 individuals had complete data for these predictors and the asthma outcome at age 10 in the IOWBC. Following a 2:1 stratified split of the data into a training and hold-out validation set, a support vector machine (SVM) algorithm was trained on the training dataset, was oversampled by 300% using an ADAptive SYNthetic (ADASYN) sampling approach and the number of controls further undersampled to balance class proportions. Hyperparameters for the SVM (with parameters kernel=linear, C=0.33) were identified through a grid search within a 5-fold cross validation framework.

Model Integration

The genomic biomarkers were integrated with the clinical models (CAPE/ CAPP) in a stepwise manner, whereby the following models were developed: i) clinical model plus PRS; ii) clinical model plus nMRS; iii) clinical model plus cMRS; iv) clinical model plus PRS and nMRS; and v) clinical model plus PRS and cMRS. The models were integrated by adding the relevant genomic risk scores as additional predictors to each clinical model's existing feature set.

The integrated CAPE and CAPP models were then retrained using the same algorithm and training dataset characteristics as identified in Kothalawala *et al.* [25], respectively. Specifically, for each integrated CAPE model, the dataset of individuals with complete data for all features was split into a training and hold-out validation set (2:1 ratio, preserving class proportions) and the training dataset was undersampled to balance class proportions. In contrast, for each integrated CAPP model, the dataset of individuals with complete data for all features was split into a training and hold-out validation set (2:1 ratio, preserving class proportions) and the number of cases in the training

dataset was oversampled by 300% using ADASYN and the number of controls further undersampled to balance class proportions. Both sets of integrated models were developed using support vector machine algorithms, with the hyperparameters for each model being tuned using a grid search (RBF kernel for the integrated CAPE model and linear kernel for the integrated CAPP models).

External validation of the genomic and integrated childhood asthma prediction models

The generalisability of the genomic risk scores and the integrated CAPE and CAPP models was assessed in the unselected MAAS cohort. Only individuals with complete data for the predictors in each model and the asthma outcome were used in the external validation analyses.

A PRS was calculated for each individual in MAAS using the SNPs included in the best PRS calculated in the IOWBC. Even if proxy SNPs were used in the IOWBC, the presence of the original SNP detailed in the curated list of 128 independent asthma SNPs was first evaluated. Where SNPs were unavailable in MAAS, proxy SNPs were sourced as prebiously detailed. If proxy SNPs were unavailable, SNPs with missing data were excluded from the cohort's PRS.

The MRSs and their subsequent integrated CAPE and CAPP models were unable to be validated in MAAS due to the unavailability of suitable methylation data samples (DNA methylation in MAAS was measured in cord blood samples using the Illumina 27K microarray).

Supplementary Figures

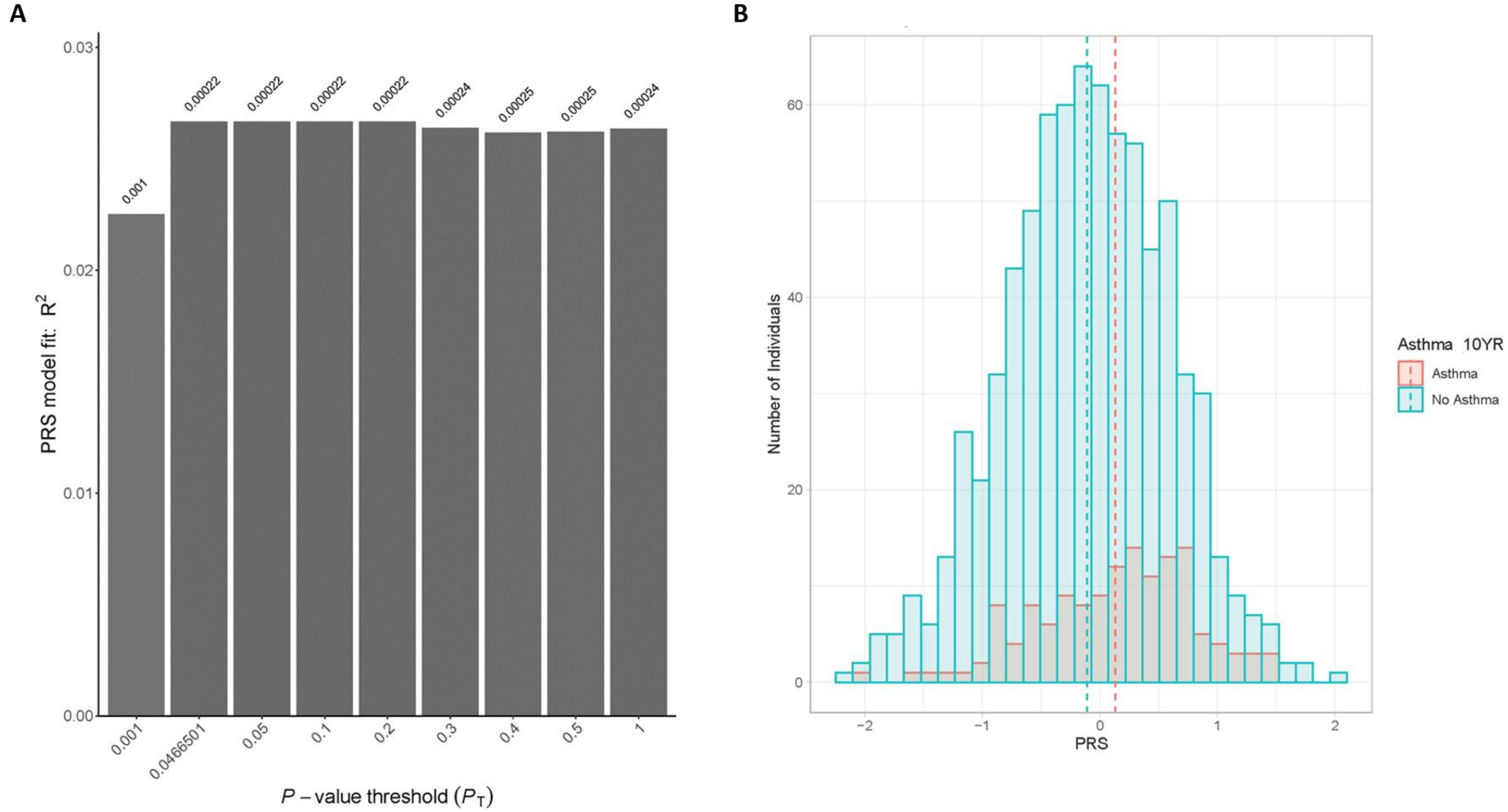

Figure S1 Evaluation of the best performing childhood asthma PRS in the IOWBC

Evaluation of PRSs constructed across a range of p-value thresholds identified a 105-SNP PRS, including SNPs with p-value less than 0.047, to explain the greatest degree of variance in the asthma phenotype. (A) Bar plot showing the model fit (Nagelkerke's R^2) for the PRSs evaluated using PRSice, constructed across a range of p-value thresholds. Values on top of each bar represents the empirical p-value of association of each PRS. (B) Histogram displaying the distribution of the PRS among asthmatic and non-asthmatic individuals in the IOWBC. Dashed lines correspond to the mean PRS for each class.

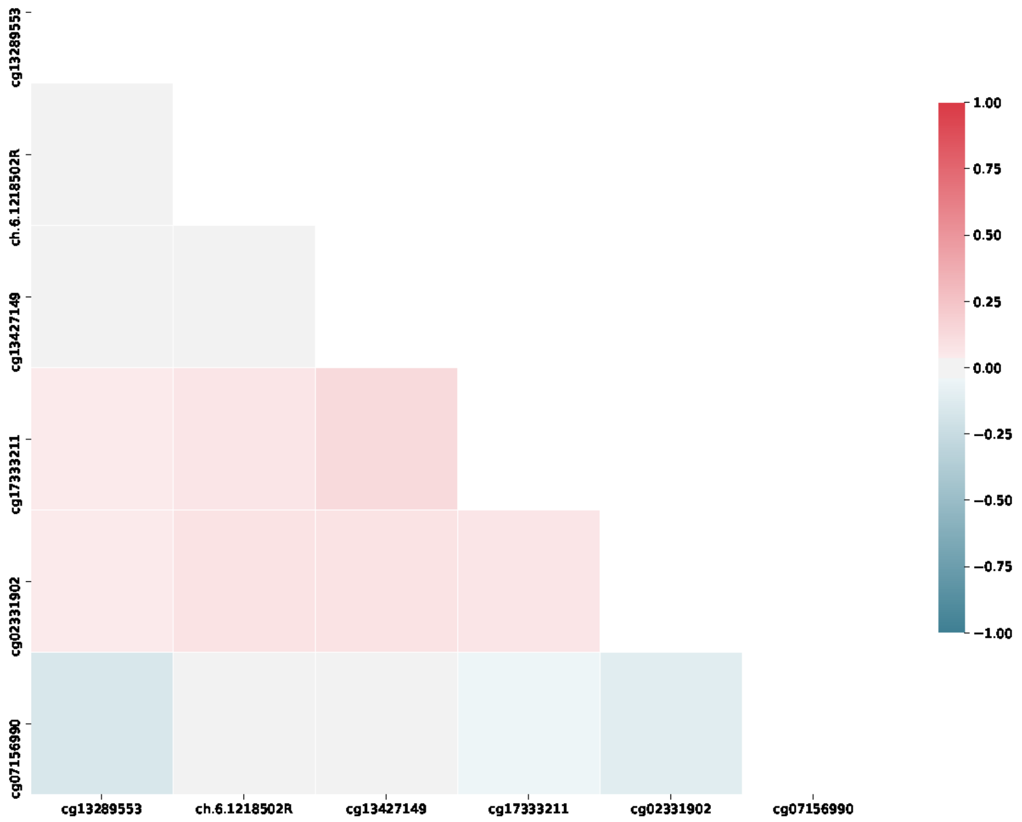

Figure S2 Correlation matrix of candidate CpGs considered for the newborn MRS

Spearman's rank correlation coefficient was used to assess the collinearity between all pairs of candidate CpGs considered for the newborn MRS. Correlation between CpGs are visualised using the colour scale, with perfect positive correlations in red and perfect negative correlations in blue.

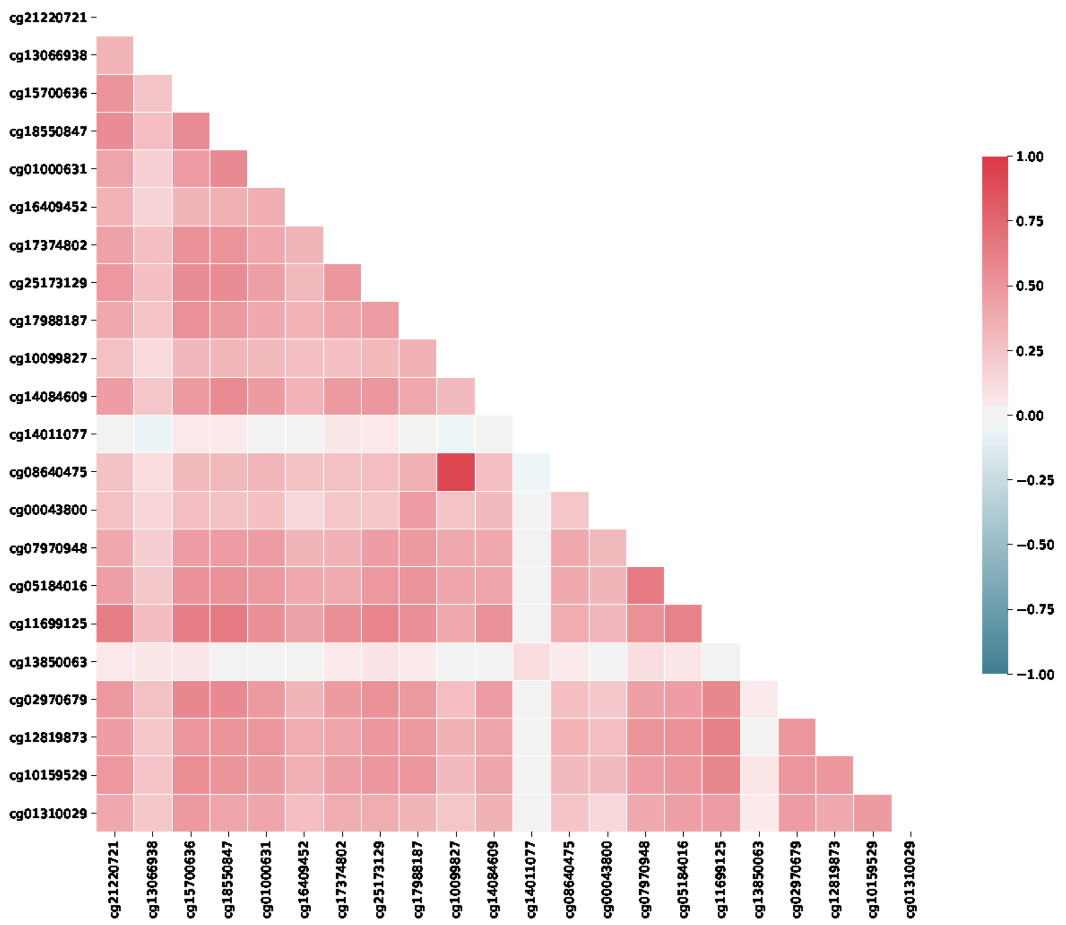

Figure S3 Correlation matrix of the 22 nearby CpGs considered for the childhood MRS

Spearman's rank correlation coefficient was used to assess the collinearity between nearby pairs of candidate CpGs considered for the childhood MRS (within 2000 base pairs of each other). Correlation between CpGs are visualised using the colour scale, with perfect positive correlations in red and perfect negative correlations in blue.

References

1. Arshad SH, Holloway JW, Karmaus W, et al. Cohort Profile: The Isle Of Wight Whole Population Birth Cohort (IOWBC). International Journal of Epidemiology 2018;47:1043-4i.
2. Asher MI, Keil U, Anderson HR, et al. International study of asthma and allergies in childhood (ISAAC): rationale and methods. European Respiratory Journal 1995;8:483-91.
3. McCarthy S, Das S, Kretzschmar W, et al. A reference panel of 64,976 haplotypes for genotype imputation. Nature Genetics 2016;48:1279-83.
4. Loh P-R, Danecek P, Palamara PF, et al. Reference-based phasing using the Haplotype Reference Consortium panel. Nature Genetics 2016;48:1443-8.
5. Durbin R. Efficient haplotype matching and storage using the positional Burrows-Wheeler transform (PBWT). Bioinformatics 2014;30:1266-72.
6. The International HapMap 3 Consortium. Integrating common and rare genetic variation in diverse human populations. Nature 2010;467:52-8.
7. Ziyab AH, Karmaus W, Yousefi M, et al. Interplay of filaggrin loss-of-function variants, allergic sensitization, and eczema in a longitudinal study covering infancy to 18 years of age. PLoS One 2012;7:e32721.
8. Beyan H, Down TA, Ramagopalan SV, et al. Guthrie card methylomics identifies temporally stable epialleles that are present at birth in humans. Genome Research 2012;22:2138-45.
9. Mukherjee N, Arathimos R, Chen S, et al. DNA methylation at birth is associated with lung function development until age 26 years. European Respiratory Journal 2021;57.
10. Lehne B, Drong AW, Loh M, et al. A coherent approach for analysis of the Illumina HumanMethylation450 BeadChip improves data quality and performance in epigenome-wide association studies. Genome Biology 2015;16:37.
11. Aryee MJ, Jaffe AE, Corrada-Bravo H, et al. Minfi: a flexible and comprehensive Bioconductor package for the analysis of Infinium DNA methylation microarrays. Bioinformatics 2014;30:1363-9.
12. Pidsley R, CC YW, Volta M, Lunnon K, Mill J, Schalkwyk LC. A data-driven approach to preprocessing Illumina 450K methylation array data. BMC Genomics 2013;14:293.
13. Leek JT, Johnson WE, Parker HS, Jaffe AE, Storey JD. The sva package for removing batch effects and other unwanted variation in high-throughput experiments. Bioinformatics 2012;28:882-3.
14. Pidsley R, Zotenko E, Peters TJ, et al. Critical evaluation of the Illumina MethylationEPIC BeadChip microarray for whole-genome DNA methylation profiling. Genome Biology 2016;17:208.
15. Custovic A, Simpson BM, Murray CS, Lowe L, Woodcock A. The National Asthma Campaign Manchester Asthma and Allergy Study. Pediatric Allergy and Immunology 2002;13:32-7.
16. Belgrave DCM, Simpson A, Semic-Jusufagic A, et al. Joint modeling of parentally reported and physician-confirmed wheeze identifies children with persistent troublesome wheezing. Journal of Allergy and Clinical Immunology 2013;132:575-83 e12.
17. El-Husseini ZW, Gosens R, Dekker F, Koppelman GH. The genetics of asthma and the promise of genomics-guided drug target discovery. The Lancet Respiratory Medicine 2020;8:1045-56.
18. Machiela MJ, Chanock SJ. LDlink: a web-based application for exploring population-specific haplotype structure and linking correlated alleles of possible functional variants. Bioinformatics 2015;31:3555-7.
19. Ferreira MAR, Mathur R, Vonk JM, et al. Genetic Architectures of Childhood- and Adult-Onset Asthma Are Partly Distinct. American Journal of Human Genetics 2019;104:665-84.
20. Myers TA, Chanock SJ, Machiela MJ. LDlinkR: An R Package for Rapidly Calculating Linkage Disequilibrium Statistics in Diverse Populations. Frontiers in Genetics 2020;11:157.
21. Choi SW, Mak TS, O'Reilly PF. Tutorial: a guide to performing polygenic risk score analyses. Nature Protocols 2020;15:2759-72.
22. Reese SE, Xu CJ, den Dekker HT, et al. Epigenome-wide meta-analysis of DNA methylation and childhood asthma. Journal of Allergy and Clinical Immunology 2019;143:2062-74.

23. Affinito O, Palumbo D, Fierro A, et al. Nucleotide distance influences co-methylation between nearby CpG sites. Genomics 2020;112:144-50.
24. Martin TC, Yet I, Tsai P-C, Bell JT. coMET: visualisation of regional epigenome-wide association scan results and DNA co-methylation patterns. BMC Bioinformatics 2015;16.
25. Kothalawala DM, Murray CS, Simpson A, et al. Development of Childhood Asthma Prediction Models using Machine Learning Approaches. medRxiv 2021.
26. Hüls A, Czamara D. Methodological challenges in constructing DNA methylation risk scores. Epigenetics 2019;15:1-11.
27. Fernández-Sanlés A, Sayols-Baixeras S, Curcio S, Subirana I, Marrugat J, Elosua R. DNA Methylation and Age-Independent Cardiovascular Risk, an Epigenome-Wide Approach. Arteriosclerosis, Thrombosis, and Vascular Biology 2018;38:645-52.
28. Guan Z, Raut JR, Weigl K, et al. Individual and joint performance of DNA methylation profiles, genetic risk score and environmental risk scores for predicting breast cancer risk. Molecular Oncology 2019;14:42-53.
29. Yu H, Raut JR, Schöttker B, Holleczek B, Zhang Y, Brenner H. Individual and joint contributions of genetic and methylation risk scores for enhancing lung cancer risk stratification: data from a population-based cohort in Germany. Clinical Epigenetics 2020;12.
30. Elliott HR, Tillin T, McArdle WL, et al. Differences in smoking associated DNA methylation patterns in South Asians and Europeans. Clinical Epigenetics 2014;6.
